# Endophytic fungi harbored in the root of *Sophora tonkinensis* Gapnep: Diversity and biocontrol potential against phytopathogens

**DOI:** 10.1002/mbo3.437

**Published:** 2017-03-15

**Authors:** Yu Qun Yao, Fang Lan, Yun Ming Qiao, Ji Guang Wei, Rong Shao Huang, Liang Bo Li

**Affiliations:** ^1^ College of AgricultureGuangxi UniversityNanningChina; ^2^ School of MedicineGuangxi University of Science and TechnologyLiuzhouChina

**Keywords:** endophytic fungal communities, geographic locality, inhibitory activity, tissue type

## Abstract

This work, for the first time, investigated the diversity of endophytic fungi harbored in the xylem and phloem of the root of *Sophora tonkinensis* Gapnep from three geographic localities with emphasis on the influence of the tissue type and geographic locality on endophytic fungal communities and their potential as biocontrol agents against phytopathogens of *Panax notoginseng*. A total of 655 fungal strains representing 47 taxa were isolated. Forty‐two taxa (89.4%) were identified but not five taxa (10.6%) according to morphology and molecular phylogenetics. Out of identifiable taxa, the majority of endophyte taxa were Ascomycota (76.6%), followed by Basidiomycota (8.5%) and Zygomycota (4.3%). The alpha‐diversity indices indicated that the species diversity of endophytic fungal community harbored in the root of *S. tonkinensis* was very high. The colonization and species diversity of endophytic fungal communities were significantly influenced by the geographic locality but not tissue type. The geographic locality and tissue type had great effects on the species composition of endophytic fungal communities. Forty‐seven respective strains were challenged by three fungal phytopathogens of *P. notoginseng* and six strains exhibited significant inhibitory activity. It was noteworthy that endophytic *Rhexocercosporidium* sp. and *F. solani* strongly inhibited pathogenic *F. solani* and other fungal phytopathogens of *P. notoginseng*.

## 1 INTRODUCTION

Endophytes are microorganisms that reside within internal tissues of living plants without visibly harming the host plant (Clay, 1992; Hyde & Soytong, 2008; Schulz & Boyle, 2005). Endophytes, mainly represented by both endophytic fungi and endophytic bacteria, have great promise with diverse potential for exploitation (Staniek, Woerdenbag, & Kayser, 2008; Strobel, Daisy, Castillo, & Harper, 2004). Recently, enormous biological diversity (Li, Zhao, Liu, & Xu, 2010; Tejesvi, Kajula, Mattila, & Pirttilä, 2011) coupled with capability to biosynthesize bioactive secondary metabolites (Aly, Debbab, Kjer, & Proksch, 2010; Chandra, 2012) and tremendous potential as biocontrol agents (Mejia et al., 2008; Zhang et al., 2014) have provided the impetus for several investigations on endophytic fungi.

The diversity of endophytic fungal communities in the tissues of plants aboveground as well as belowground, including stems, leaves, and/or roots is very high (Kusari, Kusari, Spiteller, & Kayser, 2013; Qadri, Rajput, Abdin, Vishwakarma, & Riyaz‐Ul‐Hassan, 2014). The colonization frequency (CF), species diversity, and species composition of endophytic fungal communities are affected by season, tissue type, geographic location (Mishra et al., 2012), tissue age (Nascimento et al., 2015), and host (Gonzalez and Tello, 2011; Kernaghan & Patriquin, 2015). Many endophytic fungi have the potential to synthesize secondary metabolites with various bioactivities (Kusari, Hertweck, & Spiteller, 2012), including cytotoxicity (Xu, Espinosa‐Artiles, Liu, Arnold, & Gunatilaka, 2013), antimicrobial activity (Li, Jiang, Guo, Zhang, & Che, 2008), anti‐ HIV‐1 activity (Li et al., 2008), insecticidal activity (Sappapan et al., 2008), and antioxidant activity (Wang et al., 2006), which may directly or indirectly be used as therapeutic agents against numerous diseases. Occasionally, endophytic fungi that produce host plant secondary metabolites (Kusari, Lamshoeft, Zuehlke, & Spiteller, 2008; Stierle, Strobel, & Stierle, 1993) with therapeutic value or potential have been discovered. Endophytic fungi have recently been considered an important resource for screening biocontrol agents to suppress insects and pathogens attacking plants including the host (Kusari et al., 2013; Mejia et al., 2008) and other plants (Bailey et al., 2008; Waweru, Losenge, Kahangi, Dubois, & Coyne, 2013), promoting host plant growth (Chen et al., 2010; Silva, Tozzi, Terrasan, & Bettiol, 2012).

*Sophora tonkinensis* Gapnep, is a well‐known medicinal plant of China that grows in an area of karst topography near the Tropic of Cancer, is mainly distributed in Guangxi province, and oddly is found in Guangdong province as well as Yunnan province (Wang, Xie, Fan, & Liu, 2011). The chemical constituents (Wang et al., 2011), including primarily flavonoids, alkaloids, polysaccharides, and saponins, have been isolated from the root of *S. tonkinensis*, and have pharmacological activities (Commission, 2015b; Wang et al., 2011) such as antitumor activity, antimicrobial activity, antiinflammation, antiarrhythmia, antihypertension, hepatoprotection, and immune stimulation. The crude extracts from the root of *S. tonkinensis* have been effectively applied to control symptoms on *Panax notogin*seng, a famous traditional Chinese herb with a long history in China as a valuable cardiovascular remedy (Commission, 2015a; Li, Xie, Fan, & Wang, 2011). These symptoms, namely black spots caused by *Alternaria panax* Whetz (Wei & Chen, 1992), anthracnose by *Colletotrichum gloeosporioides* (Wei, Chen, & Wu, 1989), and root rot by *Fusarium solan*i (Miao et al., 2006), seriously affect the quality and yield of *P. notoginseng* in the geo‐authentic‐producing areas. During the course of plant–endophyte coevolution, it might be possible for endophytes to assist the plant in chemical defense by producing bioactive secondary metabolites according to the theories of “mosaic effect” and “acquired immune systems” (Carroll, 1991; Rodriguez, Redman, & Henson, 2004). Hence, several members of endophytic fungi harbored in the root of *S. tonkinensis* may have an antagonistic activity against three fungal phytopathogens of *P. notoginseng*. Therefore, we selected this plant to isolate endophytic fungi. Most of the studies on endophytic fungi have been carried out in tropical, subtropical, temperate, and boreal regions, but there are only a few studies that have been carried out near the Tropic of Cancer, and overall, no major studies exist on endophytic fungi harbored in medicinal plant from karst topography near the Tropic of Cancer in Guangxi province of China.

The aim of this study was to isolate and identify endophytic fungi harbored in the root of *S. tonkinensis,* characterize the diversity of endophytic fungal communities, investigate the influence of the tissue type and geographic locality on the colonization, species diversity, and species composition of endophytic fungal communities, and further screen them for potential as biocontrol agents against three phytopathogens of *P. notoginseng* cultivated in China. The findings will not only enrich the knowledge of endophytic fungi from *S. tonkinensis* but also benefit the development of organic cultivation techniques for *P. notoginseng* in China. To the best of our knowledge, this report is the first to describe the diversity, phylogeny, and communities of endophytic fungi harbored in the root of *S. tonkinensis*, and assess their potential as biocontrol agents against phytopathogens of *P. notoginseng*.

## 2 MATERIALS AND METHODS

### 2.1 Sample collection from selected sites

In 2014, healthy plants of *S. tonkinensis* were collected in three periods from three different localities of traditional geo‐authentic‐producing areas (Wang et al., 2011) in Guangxi province of south China: Tiandeng county (T), where *S. tonkinensis* grows as a natural part of an intact shrub forest; Jingxi county (J), where *S. tonkinensis* grows in the rock crack in limestone mountainous areas; and Guangxi university (G), where *S. tonkinensis* is cultivated in a medicinal herb garden. Details of three sampling localities and dates were given in Table 1. These plants were carefully up‐rooted with the help of a spade, placed in jute bags, labeled, immediately transported to the laboratory, and processed within 24 hr of collection. Import of the plant material from Tiandeng county and Jingxi county was allowed according to the permission of the Department of Forestry of Guangxi province, Guangxi, China, and that from Guangxi university was allowed according to the permission of the College of Agriculture, Guangxi University, Guangxi, China.

**Table 1:** Location characteristics and sampling dates in this work

Sampling locations	Sampling dates	Geographic coordinates	Altitude (m)	Annual rainfall (mm)	Mean temperature (^°^C)
G: medicinal herb garden, Guangxi university	04.02.2014	23°07′8′′N,	76	1309.7	21.8
	15.05.2014	108°17′28′′E			
	02.10.2014				
T: Hongkui village, Tiandeng town, Tiandeng county	04.02.2014	23°06′36′′N,	437.1	1409.3	20.7
	15.05.2014	107°08′20′′E			
	02.10.2014				
J: Chengliang village, Xinjing town, Jingxi county	04.02.2014	23°08′49′′N,	850	1634.2	19.5
	15.05.2014	106°25′26′′E			
	02.10.2014				

### 2.2 Isolation of endophytic fungi

**Figure 1:**
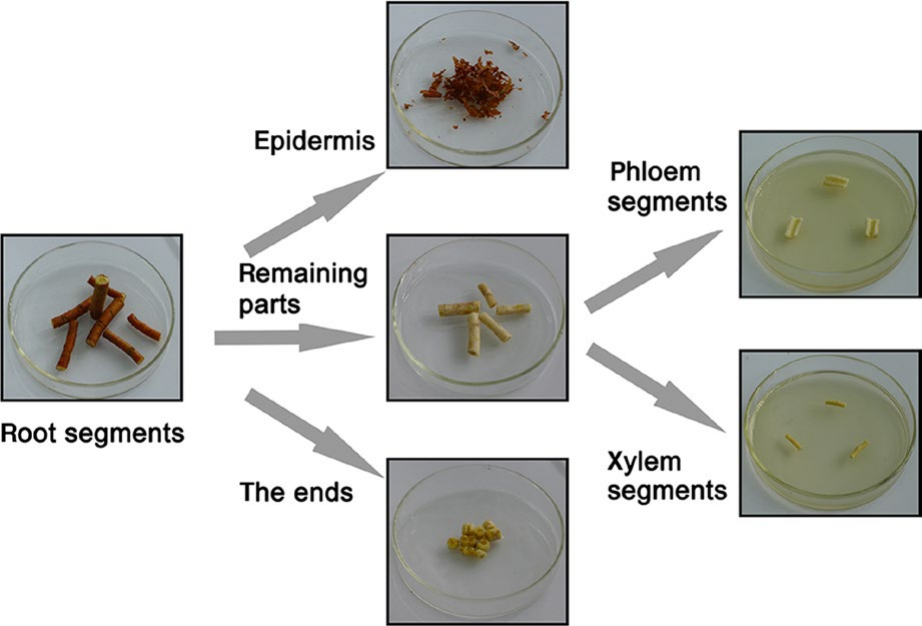
The dissection of the roots in this work

Root samples (diameter, 1–2 cm) were excised from the plant and cut into segments (length, 5–7 cm). For the surface sterilization and isolation of endophytic fungi, we established the optimum procedures according to previously described methods (Kusari et al., 2013; Tejesvi et al., 2011). The root segments were thoroughly washed in running tap water for 30 min and rinsed with double‐distilled water for 10 min. Next, the samples were sterilized with 75% ethanol for 1 min, sodium hypochlorite containing 1% available chlorine for 2 min, and 75% ethanol for 30 s. Finally, these surface‐sterilized samples were rinsed three times with sterile, double‐distilled water to remove excess surface sterilants, blotted on a sterile filter paper, and dried under aseptic conditions. To ensure the isolation of endophytic fungi, the epidermis and ends of each root segment were removed. The xylem (X) and phloem (P) were separated from the remaining part of each root segment (R) and transversely cut into 1‐cm‐long pieces, respectively, which were individually placed in Petri dishes (9 cm in diameter) containing potato dextrose agar (PDA) with chloramphenicol to eliminate any bacterial growth. The dissection of the roots was showed in Figure 1 **.** The Petri dishes with three pieces per dish were incubated at 28°C and checked daily for fungal growth for up to 6 weeks. Each colony which emerged from the segments was transferred to an antibiotic‐free PDA medium. Purification was carried out by cutting a small piece of media with mycelia at the edge of a colony and then transplanted on to new medium plates.

### 2.3 Storage of the purified endophytic fungi

Every purified endophytic fungus sample received a specific code number according to its origin (e.g., TRXY‐1 or TRXY‐2, from the xylem of the root collected from Tiandeng county, and TRPH‐1 TRPH‐2, from the phloem of the root collected from Tiandeng county). All endophytic fungi were deposited at the College of Agriculture, Guangxi University, Guangxi, China. For short‐term storage, they were cultured on PDA at 28°C for 3–10 days and maintained at 4°C (up to 3 months); for long‐term storage, they were preserved with spores or mycelia in 25% (v/v) glycerol at −80°C.

### 2.4 Genomic DNA extraction, PCR amplification, and sequencing

Endophytic fungi were cultured on sterilized cellophane stuck on PDA at 28°C for 3–10 days. Fresh cultures were harvested, and the genomic DNA was extracted following the previously described protocol (Guo, Hyde, & Liew, 2000). The ITS regions, including ITS1, 5.8S, and ITS2 regions of rDNA, were amplified with universal primer pairs ITS1 and ITS4 (White, Bruns, Lee, & Taylor, 1990). Amplification was performed in a 50‐μl reaction volume, which contained 5 μl of PCR buffer (10×), 4 μl of dNTP Mixture (each 2.5 mmol/L), 1 μl of each primer (20 pmol), 2.5 μl of template DNA (100 ng), 0.5 μl (2.5 U) of Taq DNA polymerase (TAKARA BIO INC., Japan, cat. no. R001A), and 36 μl of sterile double‐distilled water. A negative control using sterile double‐distilled water instead of template DNA was included in the amplification process. The thermal cycling program was as follows: 3 min of initial denaturation at 95°C, followed by 30 cycles of 30 s denaturation at 94°C, 30 s primer annealing at 55°C, 60 s extension at 72°C, and a final 10 min extension at 72°C. Next, 3 μl of PCR products from each PCR reaction was checked by electrophoresis on 1% (w/v) agarose gels containing SYBR Green I nucleic acid gel stain at 90 V (5 V cm^−1^) for 1.5 hr in TBE buffer (1×) and visualized under 300‐nm UV light. PCR products were purified using PCR Cleanup Filter Plates (MultiScreen^®^ PCRμ96; Millipore, USA) according to the manufacturer's protocol. Purified PCR products were directly sequenced with primer pairs as mentioned above in an ABI 3730‐XL DNA sequencer (Applied Biosystems, USA).

### 2.5 Fungal identification

The taxonomic identification of endophytic fungi isolated was based on morphology and molecular phylogenetics including phylogenetic position and similarity to reference sequences of the GenBank (Guo et al., 2000; Rivera‐Orduna, Suarez‐Sanchez, Flores‐Bustamante, Gracida‐Rodriguez, & Flores‐Cotera, 2011). When the isolates did not produce spores on PDA medium, sterile mycelia were cultured on quarter‐strength PDA medium containing sterilized fragments of the roots of *S. tonkinensis* to promote sporulation. All endophytic fungi were classified as different morphotypes according to their morphological characters. The ITS regions (ITS1‐5.8S rDNA‐ITS2) were amplified and sequenced for all of the morphotypes. Consequently, each sequence from different morphotypes as query sequence was matched against ITS sequences available in the GenBank by BLASTn search to obtain similar sequences. The most similar reference sequences with query sequence were used for phylogenetic analysis along with selected taxonomic reference sequences using MEGA version 6.0. In order to avoid using mis‐annotated sequences, the closest related strains with the most similar reference sequences from published references were deposited original cultures that were identified based on morphology and ITS sequencing. Similarities among sequences were calculated using the MatGAT v.2.01 software. The sequence was accepted at genus level when the similarity between a query sequence and a phylogenetically related reference sequence was higher than 95%, and the sequence was considered to be conspecific when that was higher than those within same genus. The strain with an ITS sequence showing a divergence greater than 5% with any entry at GenBank was considered as unidentified. These thresholds have been previously employed in other endophyte‐related studies to identify fungal taxa (Gonzalez & Luisa, 2011; Kusari et al., 2013; Sanchez, Bills, & Zabalgogeazcoa, 2008).

### 2.6 Fungal culture and extraction

Every selected endophytic fungus was cultured on a Petri dish containing PDA at 28°C for 5–10 days. The culture materials from each Petri dish were cut into small pieces and transferred to a 2‐L Erlenmeyer flask containing sterile solid medium, which included 400 g of potato, 20 g of dextrose, and 20 g of sucrose at 28°C for 30–40 days. The culture materials from the Erlenmeyer flask were successively extracted with methanol to yield crude extracts.

### 2.7 Preparation of crude extracts from the root of *S. tonkinensis*

The dried root of *S. tonkinensis* was pulverized and soaked in 1,000 ml of methanol for 2 weeks at room temperature. The organic solvent was filtered through a filter paper and evaporated to dryness under vacuum to afford crude extracts.

### 2.8 In vitro antagonistic assays of endophytes against fungal phytopathogens

In order to screen antagonistic fungi against phytopathogens of *P. notoginseng*, we took three fungal phytopathogens including *A. panax* (L), *C. gloeosporioides* (T), and *F. solani* (F) in *P. notoginseng* from the Institute of Plant Pathology, College of Agriculture, Guangxi University. The in vitro antagonistic activity of endophytic fungi against three fungal phytopathogens of *P. notoginseng* was tested using the coculture method established earlier (Kusari et al., 2013; Zhang et al., 2014). One mycelial plug (6 mm diameter) of each 10‐day‐old endophytic fungus was placed at the center of the dish containing approximately 25 ml of PDA, yielding a final depth of 4 mm. Three mycelial plugs (6 mm diameter) from three 3‐day‐old fungal phytopathogens were symmetrically placed 3 cm from the endophytic inoculant to establish a coculture as the coculture treatment. The fungal phytopathogens alone were symmetrically placed 3 cm from the center of the dish containing PDA with the crude extracts from the root of *S. tonkinensis* at the concentration of 2 mg/ml as the ecological treatment, and that without crude extracts as the growth control. All treatments and controls were run in duplicates. The cultures were incubated at 28°C. The colony growth radius of each fungal phytopathogen between its inoculation and the center of the dish in the treatment was measured when the fungal growth in the growth control had completely reached the center of the Petri dishes. The average radius of each fungal phytopathogen in the treatment was recorded as *R*_1,_ and that in the growth control was recorded as *R*_2_. The inhibition percentage of the growth of the fungal phytopathogen in the endophyte‐phytopathogen antagonism was calculated with the help of the modified formula as mentioned below:

$$\mathrm{Inhibition}\%=[\left(R_{2}-R_{1}\right)/R_{2}]\times 100$$

Fungal strains with great inhibitions against three fungal phytopathogens in the above test were selected for further testing antifungal activity of their crude extracts using the mycelial growth method (Rabea, Badawy, Steurbaut, & Stevens, 2009; Tian et al., 2015)**.** Every selected strain with antagonistic activity was cultured and extracted to yield crude extracts as mentioned above. All crude extracts were dissolved in 1% (v/v) dimethyl sulfoxide (DMSO). Appropriate volumes of the solutions of each crude extract were incorporated into the PDA medium and poured into the Petri dishes to obtain final concentrations ranging from 2 to 8 mg/ml according to the concentration of carbendazim wettable powders used in field, and each concentration was tested in triplicate. The positive control with carbendazim wettable powders was treated in the same way. The growth control without drug was maintained with 1% DMSO mixed with PDA medium. Every mycelial plug (6 mm diameter) from each 3‐day‐old fungal phytopathogen was, respectively, placed at the center of the Petri dishes. The cultures were incubated at 28°C, and the colony growth diameter of each fungal phytopathogen was measured when the fungal growth in the growth control had completely covered the Petri dishes. The radial growth of each fungal phytopathogen in the treatment measured by removing 6 mm from the growth diameter was recorded as *D*_1,_ and that in the growth control was recorded as *D*_2_. The inhibition percentage of mycelial growth was calculated with the help of the modified formula as follows:

$$\mathrm{Mycelial}\;\mathrm{growth}\;\mathrm{inhibition}\;\%=[\left(D_{2}-D_{1}\right)/D_{2}]\times \;100$$

### 2.9 Statistical analysis

The colonization frequency (CF) was expressed in percentages and calculated as the number of segments colonized by a single endophyte divided by the total number of segments examined ×100 (Mishra et al., 2012). The percentage of species composition (*S*_*i*_) was calculated as the number of taxa that belong to a specific phylum, class, or order divided by the total number of taxa in the sample (Botella and Diez, 2011). The relative species frequency (*P*_*i*_) was calculated as the number of isolates that belong to taxon *i* divided by the total number of isolates in the sample (Kusari et al., 2013). The fungal dominance was determined by Camargo's index (1/*s*), where *S* (Species richness) is the number of fungal taxa. A species was defined as dominant if *P*_*i*_ >1/*s*. The alpha‐diversity indices such as Species richness (*S*), Shannon‐Wiener index (*H*′), and Simpson's diversity index (1‐*D*) were assessed for the species diversity of the endophytic fungal communities harbored in the root of *S. tonkinensis*. The beta‐diversity indices such as Jaccard's index and Sorensen's index were calculated to compare the similarity of endophytic fungal communities regarding species composition between two localities or two tissues. The influences of geographic locality on CF, Shannon‐Wiener index, and Simpson's diversity index were analyzed by One‐Way ANOVA. The influences of tissue type on CF, Shannon‐Wiener index, and Simpson's diversity index were examined using analysis of *t* tests. The inhibition percentage of crude extracts from endophytic fungi against three phytopathogens was subjected to ANOVA to analyze their antifungal properties. The software “R version 3.2.3” was used for all the statistical analyses.

## 3 RESULTS

### 3.1 Fungal identification

A total of 655 endophytic fungal isolates were isolated from 3,740 tissue segments (1,870 xylem segments and 1,870 phloem segments from the root of *S. tonkinensis*) of 48 plant individuals. Among these, 470 isolates produced spores (conidia or sexual spores) on PDA medium. Furthermore, 118 isolates sporulated and were identified by morphology after inducing sporulation. All isolates were classified as 102 morphotypes according to morphological characters and subsequently identified as 47 fungal taxa according to phylogenetic analyses based on the ITS region sequence. Out of 47 taxa, 33 taxa (588 isolates) were identified to the species level, followed by seven taxa (46 isolates) to the genus level, one taxon (four isolates) to the order level, one taxon (one isolate) to the subclass level, and five unidentifiable taxa (16 isolates). The sequences from respective strains of 47 taxa in this work were deposited in the Genbank database. Out of 47 closest related strains, 39 strains were from published references but not eight strains. Details of these strains are summarized in Table 2.

**Table 2:** Summary of the endophytic fungi isolated from the root of *S. tonkinensis* with their respective strain numbers, GenBank accession numbers, and closest affiliations of the representative isolates in the GenBank according to ITS analysis

Strain number	Accession number	Closest related strain (accession number)	Similarity^a^ (%)	Reference
TRXY‐73	KP204265	*Cladosporium perangustum* (HM148139)	99.5	Bensch, K., et al. 2010
TRXY‐75	KP204270	*Cladosporium* sp. (KF367474)	100	Oliveira, B.R., et al. 2013
TRXY‐5	KP204304	*Phoma herbarum* (KJ188712)	99.8	Luo, J., et al. 2014
TRXY‐18‐1	KP204290	*Phoma* sp. (KC928322)	100	Choi, I.Y., et al. 2014
GRPH‐2‐1	KP204295	*Epicoccum* sp. (FJ176473)	99.6	Qi, F.H., et al. 2009
TRPH‐24	KP204307	*Alternaria alternata* (KJ957793)	100	Choi, M.S., et al. 2014
TRPH‐85	KP204310	Pleosporales sp. (GQ254682)	99.8	Unpublished
TRXY‐70	KP204312	*Rhytidhysteron* sp. (GU199428)	99	Sakalidis, M.L., et al. 2011
TRXY‐42‐2	KP204314	*Trichosporon asahii* (AF455425)	100	Buzina, W., et al. 2003
TRXY‐69	KP204316	*Fomitopsis* sp. (FJ372676)	99.3	Rungjindamai, N., et al. 2009
TRXY‐13‐2	KP204317	*Schizophyllum commune* (KF679517)	100	Chan, J.F., et al. 2014
TRXY‐41‐2	KP204318	Exobasidiomycetidae sp. (DQ682574)	100	Aime, M.C. 2007
GRXY‐7‐1	KP204319	*Mortierella alpina* (KC018229)	99.8	Wagner, L., et al. 2013
TRPH‐18‐1	KP204320	*Mucor circinelloides* (JN205949)	99.8	Walther, G., et al. 2013
TRXY‐32‐2	KP204332	*Aureobasidium pullulans* (JF439462)	99.8	Han, G., et al. 2011
TRPH‐105	KP204334	*Rhexocercosporidium* sp. (EU543257)	99.3	Unpublished
TRPH‐87	KP204336	*Rhexocercosporidium* sp. (EU543257)	100	Unpublished
TRXY‐50	KP204340	*Phialophora mustea* (JN123359)	99.6	Ban, Y., et al. 2012
TRPH‐68	KP204345	*Cryptosporiopsis radicicola* (GU062273)	99.6	Arhipova, N., et al. 2011
TRPH‐73	KP204347	*Talaromyces funiculosus* (GU183120)	100	Wicklow, D.T., et al. 2009
TRXY‐29	KP204349	*Talaromyces pinophilus* (JX684010)	100	Barnes, C.W., et al. 2012
JXRXY‐11	KP204350	*Talaromyces verruculosus* (HQ607791)	99.6	Rodrigues, A., et al. 2011
GRPH‐5‐2	KP204353	*Penicillium spinulosum* (KF646101)	100	Menkis, A., et al. 2014
JXRXY‐26	KP204356	*Penicillium toxicarium* (EF198650)	100	Serra, R., et al. 2008
TRXY‐33‐2	KP204359	*Penicillium sclerotiorum* (AY373931)	99.5	Haugland, R.A., et al. 2004
TRPH‐107	KP204360	*Penicillium coffeae* (AY742702)	98.5	Peterson, S.W., et al. 2005
TRPH‐62	KP204371	*Sagenomella* sp. (EU140821)	99.8	Unpublished
TRXY‐26	KP204372	*Aspergillus flavus* (KJ775476)	99.8	Visagie, C.M., 2014
JXRPH‐20	KP204373	*Aspergillus versicolor* (AJ937752)	100	Fomicheva, G.M., 2006
JXRXY‐5	KP204375	*Lasiodiplodia theobromae* (HM466953)	100	Sulaiman, R., et al. 2012
TRPH‐22‐1	KP204385	*Chaetomium aureum* (KF156298)	99.8	Stenstrom, E., et al. 2013
JXRPH‐21‐2	KP204387	*Phialocephala humicola* (AB671503)	100	Kiyuna, T., et al. 2012
JXRPH‐23	KP204388	*Chaetosphaeria* sp. (HQ630994)	98.3	Shrestha, P., et al. 2011
TRPH‐35	KP204396	*Colletotrichum simmondsii* (JN121206)	99.6	Faedda, R., et al. 2011
TRXY‐63	KP204398	*Purpureocillium lilacinum* (EU553316)	99.6	Inglis, P.W. and Tigano, M.S. 2006
TRPH‐89	KP204400	*Trichoderma asperellum* (GU198311)	100	Samuels, G.J., et al. 2010
TRPH‐13	KP204401	*Trichoderma* sp. (KF367564)	99.8	Oliveira, B.R., et al. 2013
JXRPH‐2‐1	KP204402	*Hypocrea nigricans* (JN943371)	100	Schoch, C.L., et al. 2012
GRPH‐0	KP204404	*Myrothecium verrucaria* (HQ608048)	99.0	Rodrigues, A., et al. 2011
TRXY‐58	KP204405	*Metarhizium anisopliae* (FJ545312)	99.6	Freed, S., et al. 2011
TRXY‐34‐1	KP204422	*Fusarium solani* (AB498917)	100	Hamada, N., et al. 2010
GRXY‐1	KP204407	*Fusarium oxysporum* (KJ909935)	100	Garibaldi, A., et al. 2014
TRXY‐60	KU862685	*Pleosporales sp*. (JN116643)	92.7	Supaphon, P., et al. 2014
TRXY‐56‐1	KU862686	*Trichosporon asahii* (KM982986)	92.2	Unpublished
TRPH‐94	KU862687	*Dothideomycetes sp*. (JQ905832)	91.4	Unpublished
JXRPH‐21‐1	KU862688	*Nectria haematococca* (JX868649)	81.5	Unpublished
JXRPH‐24	KT935174	*Fusarium solani* (EU982942)	80.9	Unpublished

a The similitude percentages based on ITS sequence between respective strains and closest related strains were calculated using MatGAT v. 2.01 software.

### 3.2 Phylogeny and fungal diversity analysis

**Figure 2:**
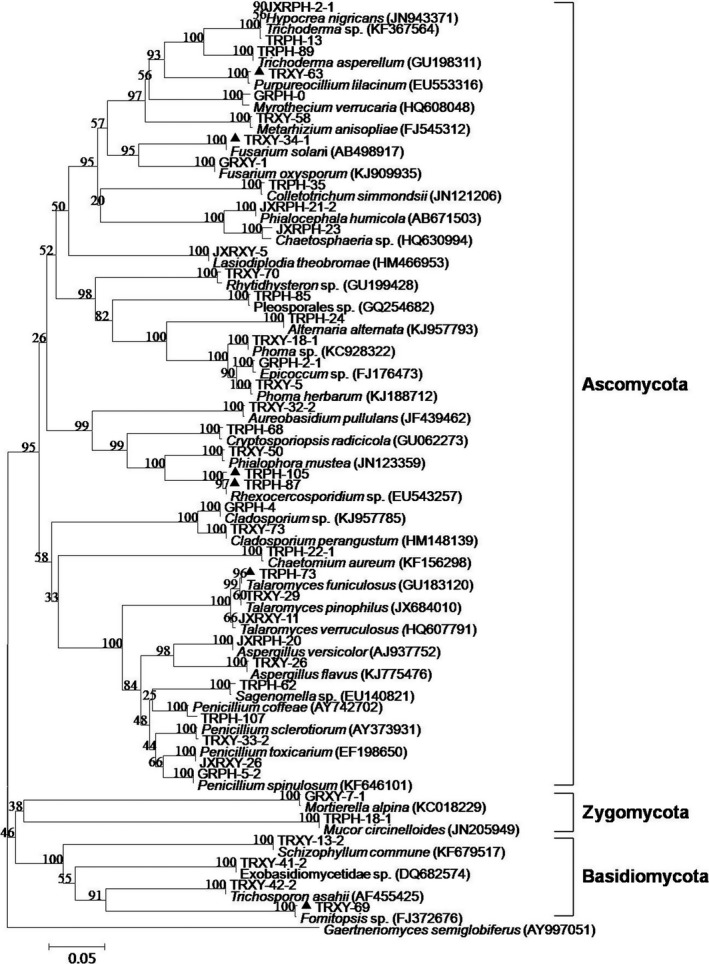
Phylogenetic tree of identifiable endophytes harbored in the root of *S. tonkinensis* based on ITS sequences using the software of MEGA version 6.0

A total of 84 ITS sequences of respective strains including 42 identifiable taxa isolated from the root of *S. tonkinensis*, 41 closely related strains, and one external reference strain retrieved from GenBank, were employed to construct the phylogenetic tree using the software of MEGA version 6.0. The respective strains with strong inhibitory activity against fungal phytopathogens were marked with a triangle **(▲).** For the closely related strains, the numbers behind the scientific name were the accession number of GenBank**.** The evolutionary history was inferred using the neighbor‐joining method. The optimal tree with the sum of branch length = 4.62712089 is shown. The percentage of replicate trees in which the associated taxa clustered together in the bootstrap test (1,000 replicates) is shown above the branches. The tree is drawn to scale, with branch lengths in the same units as those of the evolutionary distances used to infer the phylogenetic tree. The evolutionary distances were computed using the p‐distance method and are in the units of the number of base differences per site. All ambiguous positions were removed for each sequence pair. There were a total of 728 positions in the final dataset. The phylogenetic tree displayed diverse taxonomic affinities among identifiable taxa (Figure 2). All taxa were distributed in three clusters, namely phylum Ascomycota within four classes represented by 13 orders, phylum Basidiomycota with three classes represented by three orders, and phylum Zygomycota represented by two orders.

To characterize the colonization, species diversity, and species composition of endophytic fungal community in the root of *S. tonkinensis*, we calculated the total colonization frequency (CF, 17%), alpha‐diversity indices such as species richness (*S*, 47), Shannon‐Wiener index (*H*′, 3.2356), and Simpson's diversity index (*1‐D*, 0.9458), and the percentage of species composition (*S*_*i*_) (Table 3). The species richness consisted of frequent species (29 species, 61.7%) and rare species (18 species, 38.3%) with singleton and doubleton isolates (Table 3 **)**, indicating that frequent species were dominant in this community. The alpha‐diversity indices showed that the species diversity was rather high in this community. According to the percentages of species composition in this community, the most abundant phylum, by far, was Ascomycota (36 taxa, 76.6%) with abundant class Sordariomycetes (12 taxa, 25.5%), Eurotiomycetes (11 taxa, 23.4%), and Dothideomycetes (10 taxa, 21.3%), represented by particular abundant order Eurotiales (10 taxa, 21.3%), Hypocreales (8 taxa, 17%), and Pleosporales (5 taxa, 10.6%). However, rare species belonged to the phylum Basidiomycota (4 taxa, 8.5%) and Zygomycota (2 taxa, 4.3%). There were 17 taxa obtained in this community that could be considered as dominant species (Table 3). The dominant species *F. solani* (0.1300) and *F. oxysporum* (0.1023) were isolated from the root of *S. tonkinensis* from three localities of Guangxi province. The next dominant species were *C. perangustum* (0.0611)*, T. pinophilus* (0.0550), *Cladosporium* sp. (0.0550), *P. coffeae* (0.0504), *M. verrucaria* (0.0473), and a collective group of species (0.0229 to 0.0427).

**Table 3:**
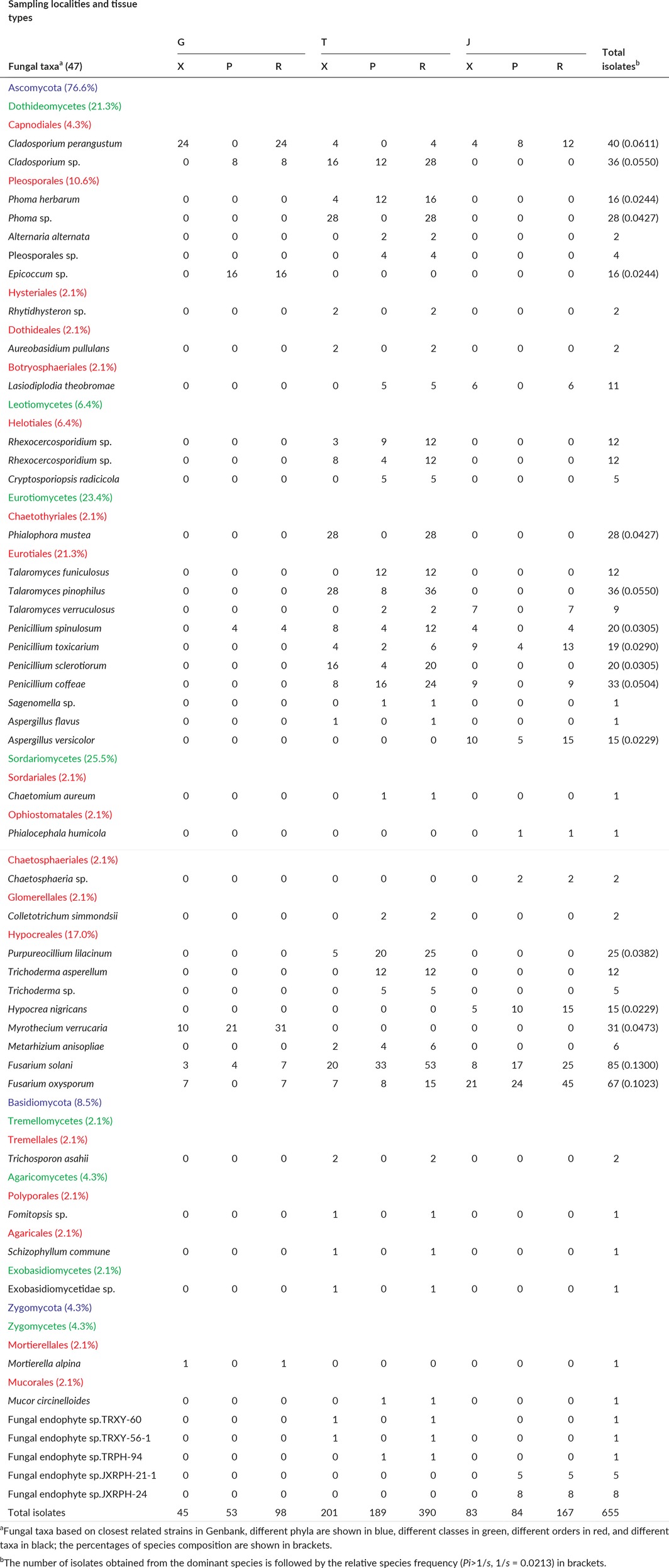
Summary of the endophytic fungi isolated from the xylem and phloem of the root of *S. tonkinensis* from three sampling localities with their taxa and the number of isolates from each taxon

### 3.3 The influences of geographic locality and tissue type on endophytic fungal communities

The influences of geographic locality and tissue type on the colonization, species diversity, and species composition of the endophytic fungal communities were investigated. The xylem and phloem of the root had no significant influence on the CF (Sig = 0.886, 0.886 >0.05), Shannon‐Wiener index (Sig = 0.941, 0.941 >0.05), and Simpson's diversity index (Sig = 0.383, 0.383 >0.05) according to *t* test, but geographic locality significantly affected these as displayed in Table 4. These results indicated that the colonization and species diversity of these communities were significantly influenced by geographic locality but not tissue type.

**Table 4:** The influences of geographic locality on CF, Shannon‐Wiener index, and Simpson's diversity index

Geographic locality^a^	CF%	Shannon‐Wiener index (*H′*)	Simpson's diversity index (1‐*D*)
G	8.0000 ± 1.3528 a	1.7170 ± 0.1226 a	0.7894 ± 0.0248 a
T	31.0667 ± 1.8148 b	3.0090 ± 0.1003 b	0.9367 ± 0.0070 b
J	13.3333 ± 1.4572 c	2.2279 ± 0.0717 c	0.8621 ± 0.0053 c

CF, colonization frequency

a Data were analyzed by one‐way ANOVA followed by LSD test; results are expressed as the mean ± *SD*. (*n* = 3); and results followed by different letters are significantly different according to LSD test (*p* < .05).

Sorenson's and Jaccard's similarity indices of the endophytic fungal communities between two tissues or two localities were rather low, as exhibited in Table 5. The most dominant species was also diverse in different tissue types or geographic localities—that is, *F. oxysporum* in the xylem, *F. solani* in the phloem, *M. verrucaria* in Guangxi University, *F. solani* in Tiandeng county, and *F. oxysporum* in Jingxi county (Table 3). In addition, 16 fungal taxa exclusively colonized the phloem of the root, and 12 fungal taxa were only isolated from the xylem of that, providing clear evidence for tissue specificity (Table 3). These results revealed that geographic locality and tissue type had great effects on the species composition of these communities.

**Table 5:** Sorenson's and Jaccard's similarity indices of endophytic fungi communities between two tissues or two localities

	Tissues	Localities		
Similarity indices^a^	X and P	G and T	G and J	T and J
Sorenson's index (QS)	0.36	0.16	0.24	0.25
Jaccard's index (JS)	0.22	0.09	0.14	0.15

a Both indices range from 0 (no overlap between communities) to 1 (total overlap between communities).

### 3.4 In vitro antagonistic assays of endophytic fungi against fungal phytopathogens of *P. notoginseng*

**Figure 3:**
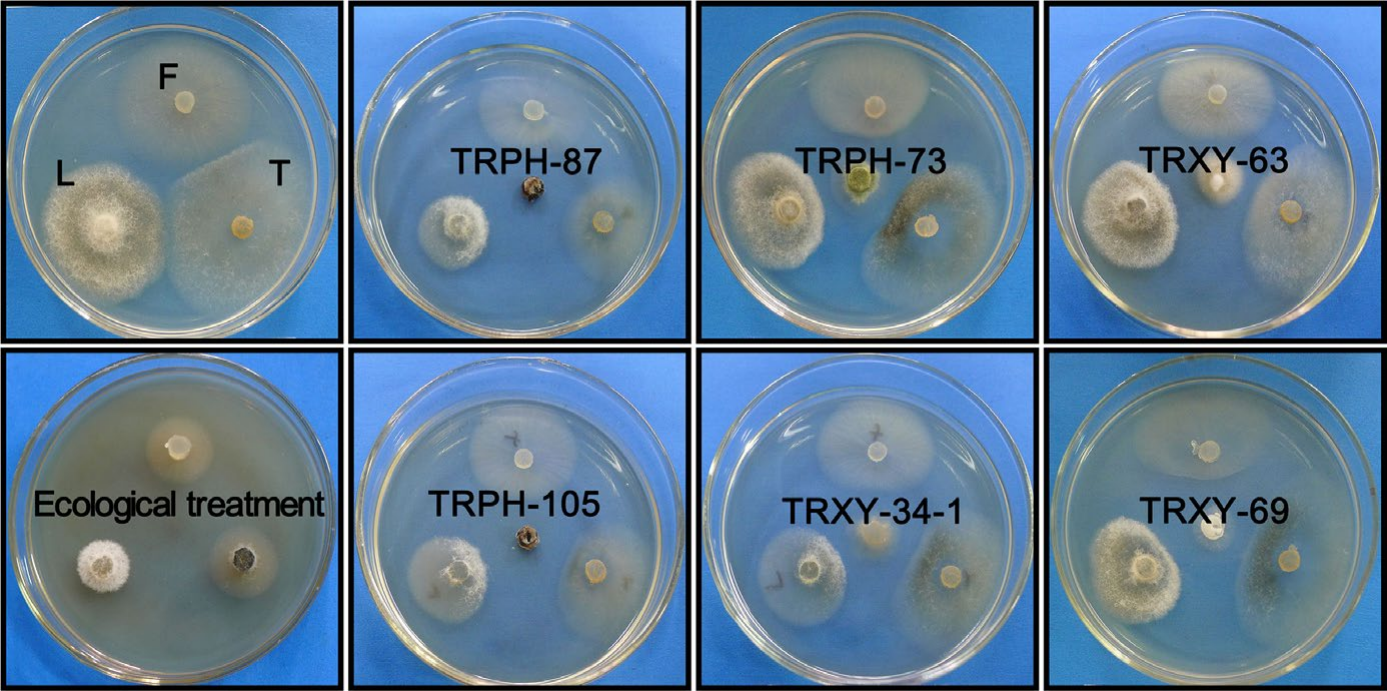
The coculture interactions between endophytic fungi strains and three fungal phytopathogens on PDA

All respective strains from 47 taxa isolated from the root of *S. tonkinensis* were screened for antagonistic activity against three fungal phytopathogens of *P. notoginseng* using the coculture method. In the ecological treatment, the crude extract from the root of *S. tonkinensis* at the concentration of 2 mg/ml showed 58% inhibition against *F. solani*, 59% inhibition against *C. gloeosporioides*, and 68% inhibition against *A. panax* (Figure 3 **)**. In coculture, 24 strains showed 50% or more inhibition against *F. solani* (15 strains)*, C. gloeosporioides* (22 strains)*,* and *A. panax* (12 strains), respectively (Table 6).

**Table 6:** Growth inhibition of fungal phytopathogens antagonized by endophytic fungi isolated from the root of *S. tonkinensis* on PDA using the coculture method

Endophytic fungi strain number	Growth inhibition of fungal phytopathogens^a^		
	*F. solani*	*C. gloeosporioides*	*A. panax*
TRXY‐75	+++	+++	+
TRXY‐5	+++	++++	++
TRXY‐18‐1	+++	++++	++++
GRPH‐2‐1	++	+++	+
TRPH‐24	++	+++	+
TRXY‐69	++++	++++	++++
TRXY‐13‐2	+++	+++	++
GRXY‐7‐1	++	+++	+
TRPH‐18‐1	+	+++	+
TRPH‐105	++++	++++	++++++
TRPH‐87	++++	++++	++++
TRPH‐68	+	+++++	++
TRPH‐73	++++	++++	++++
TRXY‐29	++	+++	++
JXRXY‐26	+++	++	++
TRXY‐33‐2	++++	++++	+++
TRXY‐26	++++	+++	++++
JXRXY‐5	+++	+	+++
TRPH‐22‐1	++	++++	++++
TRXY‐63	++++	++++	++++
TRPH‐89	++	++++	++++
TRPH‐13	+	+++	++
TRXY‐34‐1	++++	+++++	++++
GRXY‐1	+++	+++	++

a Symbols represent inhibition percentage in different ranges: +, <40%; ++, 40%–49%; +++, 50%–59%; ++++, 60%–69%; +++++,70%–79%; ++++++, >80%.

**Figure 4:**
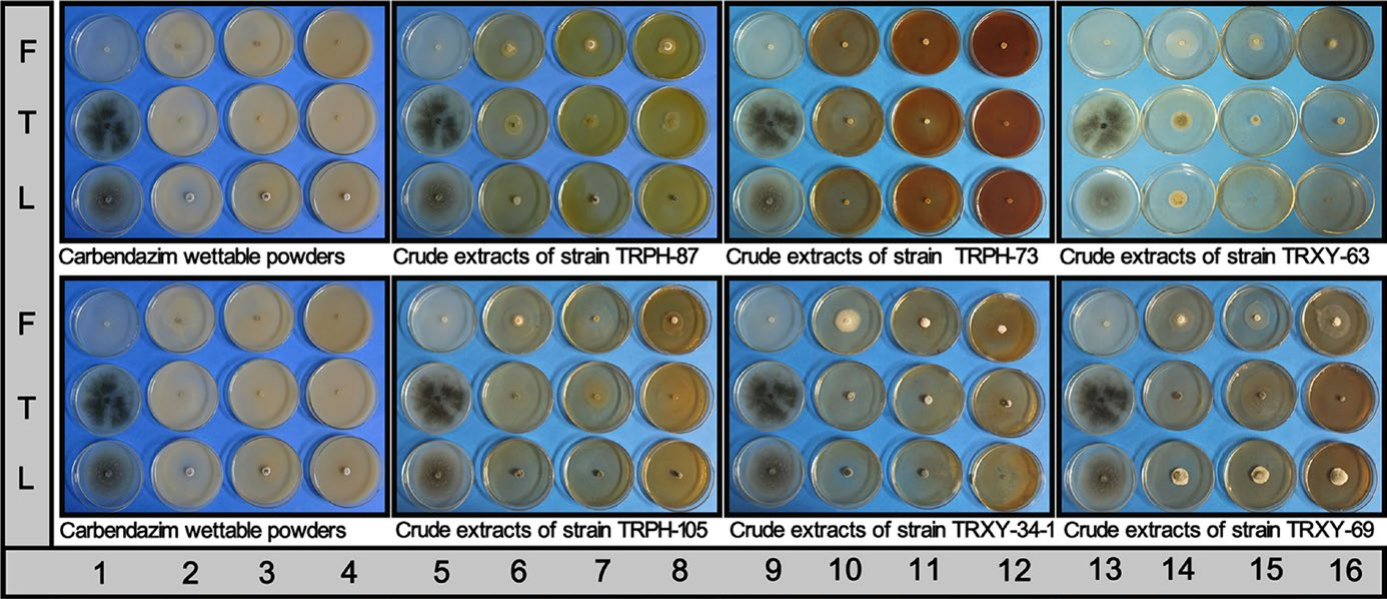
The mycelial growth of fungal phytopathogens on PDA‐containing drug. Notes: growth control (columns: 1, 5, 9, 13), treated dishes (columns: 2–4, 6–8, 10–12, 14–16)

Six endophytic strains with more than 60% inhibition against all of three fungal phytopathogens were selected to further test the antifungal activity of their crude extracts using the mycelial growth method (Table 7, Figure**** 3, Figure 4)**.** In the test plates, mycelial growth inhibition, including no growth, only growth on the mycelial plug and growth on the medium, was observed. The colonial morphology was changed in the plates with crude extracts from different strains. Crude extracts from strains TRPH‐73, TRPH‐105, and TRPH‐87 exhibited more than 90% inhibition against all of three fungal phytopathogens even at the low concentration of 2 mg/ml. The most susceptible phytopathogen was *C. gloeosporioides* whose mycelial growth was completely inhibited by the crude extracts of strains TRPH‐73, TRPH‐87, and TRPH‐105, even at the low concentration of 2 mg/ml, and by the crude extracts of strains TRXY‐34‐1, TRXY‐69, and TRXY‐63 at the concentration of 8 mg/ml. The six strains showed significant antifungal activity against three fungal phytopathogens, based on that of carbendazim wettable powders, which were widely applied to control fungal phytopathogens of *P. notoginseng* using the concentration range of 2–8 mg/ml. Particularly, the antifungal activity of the crude extracts from strains TRPH‐73, TRPH‐105, TRPH‐87, and TRXY‐34‐1 was more than that of carbendazim wettable powders against *A. panax* and almost equal to that of carbendazim wettable powders against *C. gloeosporioides*. The inhibitory activity of the crude extracts from strains TRPH‐73 and TRPH‐105 was also equal to that of carbendazim wettable powders against *F. solani*.

**Table 7:** Percent of inhibitory activity on mycelial growth of fungal phytopathogens produced by the crude extracts of six endophytic strains from the root of *S. tonkinensis* on PDA

		Fungal phytopathogens^b^		
Treatment^a^	Concentration mg/ml	*F. solani*	*C. gloeosporioides*	*A. panax*
Carbendazim wettable powders	2	100.00 ± 0.00	100.00 ± 0.00	93.17 ± 0.35
	4	100.00 ± 0.00	100.00 ± 0.00	94.17 ± 0.47
	8	100.00 ± 0.00	100.00 ± 0.00	96.10 ± 0.36
Crude extracts of strain TRPH‐73	2	100.00 ± 0.00	100.00 ± 0.00	100.00 ± 0.00^c^
	4	100.00 ± 0.00	100.00 ± 0.00	100.00 ± 0.00^c^
	8	100.00 ± 0.00	100.00 ± 0.00	100.00 ± 0.00^c^
Crude extracts of strain TRPH‐105	2	90.10 ± 0.36^c^	100.00 ± 0.00	96.20 ± 0.44^c^
	4	99.9 ± 0.10	100.00 ± 0.00	97.23 ± 0.49^c^
	8	99.83 ± 0.21	100.00 ± 0.00	99.03 ± 0.25^c^
Crude extracts of strain TRPH‐87	2	93.93 ± 0.40^c^	100.00 ± 0.00	99.20 ± 0.44^c^
	4	94.20 ± 0.44^c^	100.00 ± 0.00	99.03 ± 0.15^c^
	8	94.06 ± 0.40^c^	100.00 ± 0.00	100.00 ± 0.00^c^
Crude extracts of strain TRXY‐34‐1	2	68.10 ± 0.66^c^	94.07 ± 0.40^c^	93.13 ± 0.42
	4	91.10 ± 0.36^c^	97.17 ± 0.38^c^	94.17 ± 0.47
	8	94.90 ± 0.46^c^	100.00 ± 0.00	100.00 ± 0.00^c^
Crude extracts of strain TRXY‐69	2	88.10 ± 0.36^c^	96.13 ± 0.32^c^	78.03 ± 0.45^c^
	4	95.27 ± 0.71^c^	99.10 ± 0.36^c^	78.07 ± 0.21^c^
	8	95.23 ± 0.31^c^	100.00 ± 0.00	78.20 ± 0.41^c^
Crude extracts of strain TRXY‐63	2	78.10 ± 0.32^c^	94.27 ± 0.21^c^	67.93 ± 0.40^c^
	4	85.43 ± 0.25^c^	96.53 ± 0.31^c^	80.30 ± 0.40^c^
	8	91.50 ± 0.20^c^	100.00 ± 0.00	84.30 ± 0.56^c^

a Carbendazim wettable powders containing 50% carbendazim.

b Results are expressed as the mean ± S.D. (*n* = 3); data were analyzed by one‐way ANOVA followed by LSD test.

c Significant difference between each treatment and the positive control at the same concentration are shown as *p* < .05.

## 4 DISCUSSION

Morphological characteristics and ITS sequences analysis have been employed for the identification of endophytic fungi in this work. However, this work still failed to identify some taxa based on >5% divergences of ITS sequences and no spores. These unidentifiable taxa require the analysis of other gene markers to provide better taxonomic resolution. Many other markers which have been used for fungal identification are 28S rDNA gene, cytochrome c oxidase subunit I, and beta‐tubulin 2 gene (Liu, Xu, & Guo, 2007; Rivera‐Orduna et al., 2011; Robideau et al., 2011). Some endophytic fungi belonging to unidentifiable taxa may represent novel species. The taxonomic novelty of endophytic fungi may also correspond to chemical novelty of their secondary metabolites (Kumar et al., 2013). Furthermore, these endophytic fungi have not been explored for their natural products. Thus, these organisms will be given priority to isolate and characterize novel molecules from their secondary metabolites.

The total CF of endophytic fungi with 17% in the root of *S. tonkinensis* was much lower than that with the range of 33% to 53% in the roots of other medicinal plants (Jin et al., 2013; Kharwar, Verma, Strobel, & Ezra, 2008; Mishra et al., 2016). Two reasons may account for the high CF in the roots of medicinal plants in previous reports. One likely reason is that the soil fungi and rhizospheric fungi are so prevalent and diversified to easily establish an endophytic relationship with the roots (Ghimire, Charlton, Bell, Krishnamurthy, & Craven, 2011). The other reason is that roots as important sources of easily accessible substrate may provide a relatively stable environment favoring many fungal survival and coexistence (Angelini et al., 2012; Garbeva, Veen, & Elsas, 2004). However, the low CF in the root of *S. tonkinensis* may be associated with the presence of antimicrobial chemicals as mentioned above that may have suppressed the growth of some endophytic fungi.

In the root of *S. tonkinensis*, the majority of endophyte taxa were Ascomycota, a finding that was in agreement with that of previous reports (Qadri et al., 2014; Rivera‐Orduna et al., 2011; Vieira et al., 2014). The low proportion of the phylum Basidiomycota and Zygomycota were consistent with that reported in other studies (Gonzalez & Luisa, 2011; Sánchez, Bills, Acuña, & Zabalgogeazcoa, 2010; Tejesvi et al., 2011). However, recent papers have suggested that Basidiomycota constitute an important component of certain endophytic communities (Pinruan et al., 2012; Rungjindamai, Pinruan, Hattori, & Choeyklin, 2008). The abundant classes Sordariomycetes, Eurotiomycetes, and Dothideomycetes, were similar to that of endophytic fungal community associated with ferns in Costa Rica (Del Olmo‐Ruiz & Arnold, 1991) and *Huperzia serrata* in China (Xiong et al., 2015). The abundant orders Eurotiales, Hypocreales, and Pleosporales, were in line with that of endophytic fungal community in *Ficus* tree (Solis, Edison Dela Cruz, Schnittler, & Unterseher, 2013a), *Annona squamosa* (Lin et al., 2010), and *Stellera chamaejasme* L. (Jin et al., 2013), respectively. The species *F. solani*,* F. oxysporum, C. perangustum*,* Cladosporium* sp., *T. pinophilus*,* P. coffeae*, and *M. verrucaria* were dominant in this work, possibly due to the high spore production of these fungi and their cosmopolitan nature, which statistically increases their chance to become established as endophytes, as indicated in previous studies (Mishra et al., 2012; Raviraja, 2005; Schulthess & Faeth, 1998). In addition, based on the “balanced antagonisms” hypothesis (Schulz, Haas, Junker, Andree, & Schobert, 2015; Schulz, Rommert, Dammann, Aust, & Strack, 1999), they as dominant species might not only secrete toxic metabolites to inhibit microbial competitors (Breinholt et al., 1997; Lee & Lee, 2012; Zhai et al., 2015) but also possess the ability to resist the attack of the host alkaloids with antitumor and antifungal activities (Liu et al., 2014; Yang, Zhao, & Ju, 2008).

The alpha‐diversity indices indicated that the species diversity of the endophytic fungal community harbored in the root of *S. tonkinensi*s from three localities of Guangxi province was very high, showing a similarity to that in other plant hosts (Garcia, Rhoden, Rubin Filho, Nakamura, & Pamphile, 2012; Li et al., 2010). Furthermore, this community was dominated by frequent species, following the same pattern as those in other plant hosts (Gonzalez & Luisa, 2011; Kusari et al., 2013). The rare species usually were recognized as the result of unstable associations that possibly only occurred when a given plant and fungal phenotype were confronted (Joshee, Paulus, Park, & Johnston, 2009; Orlandelli, Alberto, Rubin Filho, & Pamphile, 2012). However, 38.3% rare species in this work imply that some members of these fungi are host‐specific and occupy specific ecological niche in this community (Yuan et al., 2011).

Geographic locality significantly affected the colonization, species diversity, and species composition of endophytic fungal communities harboring the root of *S. tonkinensis*, possibly because ecological environment primarily including temperature, rainfall, altitude, and geographic coordinates are diverse in three geographic localities as mentioned above. In different ecosystems, the fungi are subjected to different selection pressures (Goere & Bucak, 2007; Petrini, Sieber, Toti, & Viret, 1993). Furthermore, in order to adapt to the ecological environment, a plant may produce several toxic metabolites toward which biotransformation abilities of many endophytic fungi to a certain extent decide the colonization range of their hosts (Saunders & Kohn, 2009; Wang & Dai, 2011; Zikmundova, Drandarov, Bigler, Hesse, & Werner, 2002). These lead to the establishment of a quite specific endophytic fungal community at each geographic locality, as reported previously (Goere & Bucak, 2007; Hoffman & Arnold, 2008).

Tissue type, including root, stem, bark, twig, leaf, and seed, influenced the colonization, species diversity, and species composition of endophytic fungi communities as indicated in previous reports (Gonzalez & Luisa, 2011; Mishra et al., 2012; Raviraja, 2005). However, there were few works about endophytic fungi communities in the xylem and the phloem of the root tissue in previous studies. In this work, results showed that the xylem and phloem of the root influenced the species composition of the endophytic fungi communities but not the colonization and species diversity of that. The striking difference in the species composition of fungal communities between the xylem and phloem may be due to tissue specificity as reported in other tissues (Mishra et al., 2012; Raviraja, 2005). These tissues may represent two distinct microenvironments including toxic metabolites, oxygen, nutrition, anatomy, and endophytic bacteria consequently shaping their difference in species composition (Huang, Cai, Hyde, Corke, & Sun, 2008; Qadri et al., 2014; Schulz et al., 2015). Further work is needed to investigate the reasons for similarity in the colonization and species diversity of fungal communities between the xylem and the phloem.

This work also demonstrated that geographic locality affected the endophytic fungi communities harbored in the root of *S. tonkinensi*s more strongly than the tissue type, a finding that was not in agreement with a previous report (Mishra et al., 2012).

Some endophytic fungi with strongly antimicrobial activities as biological agents are of increasing public interest (Bailey et al., 2008; Rubini et al., 2005). Because the crude extracts from the roots of *S. tonkinensis* were effectively used to control symptoms on *P. notoginseng* cultivated in Guangxi province, we attempted to screen antagonistic fungi from endophytic fungi isolated from them against three fungal phytopathogens of *P. notoginseng*.

The results that 24 strains showed 50% or more inhibition against three fungal phytopathogens of *P. notoginseng*, suggested that it is possible to effectively screen potential biocontrol agents against fungal phytopathogens of *P. notoginseng* from the root of *S. tonkinensis*. Furthermore, the endophytic fungi and the host plant exerting similar antifungal activities proved that endophytic fungi may assist the host plant in chemical defense.

The antifungal activity of the crude extracts from six strains was more than or almost equal to that of carbendazim wettable powders against three fungal phytopathogens of *P. notoginseng* in vitro, therefore, future investigations will be conducted to study their potential as biocontrol agents on an agronomic scale.

It was noteworthy that there was a few works in the antagonistic activity and compounds of *Rhexocercosporidium* species in previous study. Therefore, the strains *Rhexocercosporidium* sp TRPH‐87 and *Rhexocercosporidium* sp TRPH‐105 probably produce new natural compounds with antifungal activity, and the isolation and characterization of the active substance from them are in progress.

The result that the endophytic strain *F. solani* TRX‐34‐1 strongly inhibited pathogenic *F. solani* compelled reconsidering whether *F. solani* TRX‐34‐1 was capable of producing associated plant secondary metabolites as a result of horizontal gene transfer (Gogarten & Townsend, 2005) from host plant to endophytic fungus during the course of evolution. In this work, *F. solani* TRX‐34‐1 is likely a nonpathogenic strain based on its antagonistic activity against three fungal phytopathogens of *P. notoginseng*. Thus, key research on the mode of action of *F. solani* TRX‐34‐1 against phytopathogens of *P. notoginseng* by several methods is progress.

In conclusion, endophytic fungal communities harbored in the roots of *S. tonkinensis* with high diversity were affected by geographic locality more strongly than tissue type, and they have great promise not only as potential sources of bioactive secondary metabolites, but also as biocontrol agents against fungal phytopathogens of *P*. *notoginseng* and possibly other pathogens.

## CONFLICT OF INTEREST

None declared.
